# Culture‐independent analysis of hydrocarbonoclastic bacterial communities in environmental samples during oil‐bioremediation

**DOI:** 10.1002/mbo3.630

**Published:** 2018-04-15

**Authors:** Narjes Dashti, Nedaa Ali, Samar Salamah, Majida Khanafer, Ghada Al‐Shamy, Husain Al‐Awadhi, Samir S. Radwan

**Affiliations:** ^1^ Microbiology program Department of Biological Sciences Faculty of Science Kuwait University Safat Kuwait

**Keywords:** bioremediation, hydrocarbonoclastic bacteria, molecular analysis, *nifH*‐genes, oil spills

## Abstract

To analyze microbial communities in environmental samples, this study combined Denaturing Gradient Gel Electrophoresis of amplified 16S rRNA‐genes in total genomic DNA extracts from those samples with gene sequencing. The environmental samples studied were oily seawater and soil samples, that had been bioaugmented with natural materials rich in hydrocarbonoclastic bacteria. This molecular approach revealed much more diverse bacterial taxa than the culture‐dependent method we had used in an earlier study for the analysis of the same samples. The study described the dynamics of bacterial communities during bioremediation. The main limitation associated with this molecular approach, namely of not distinguishing hydrocarbonoclastic taxa from others, was overcome by consulting the literature for the hydrocarbonoclastic potential of taxa related to those identified in this study. By doing so, it was concluded that the hydrocarbonoclastic bacterial taxa were much more diverse than those captured by the culture‐dependent approach. The molecular analysis also revealed the frequent occurrence of *nifH*‐genes in the total genomic DNA extracts of all the studied environmental samples, which reflects a nitrogen‐fixation potential. Nitrogen fertilization is long known to enhance microbial oil‐bioremediation. The study revealed that bioaugmentation using plant rhizospheres or soil with long history of oil‐pollution was more effective in oil‐removal in the desert soil than in seawater microcosms.

## 1 INTRODUCTION

About 0.08% to 0.4% of the crude oil produced and used on our planet is spilled as pollutants in the marine ecosystem (National Research Council, 1989). Today, those estimates undoubtedly must have increased dramatically. Terrestrial and atmospheric ecosystems are also suffering from hydrocarbon pollution. This problem is particularly acute in oil‐producing countries such as those in the Arabian/Persian Gulf region. Based on their relatively slow biodegradability, oil‐pollutants persist long in the environment (Andreoni & Gianfreda, 2007). Aromatic constituents are hazardous to living beings including humans (Gu, Kralovec, Christensen, & Van Camp, 2003; Sverdrup, Nielsen, & Krogh, 2002). That is why more research in the field of controlling environmental oil‐pollution is still needed. Spilled oil is removed, either by physical and chemical means, for example, land‐filling and incineration (Kuiper, Lagendijk, Bloemberg, & Lugtenberg, 2004) or using catabolic activities of microorganisms, that is, bioremediation (Atlas, 1991; Atlas & Bartha, 1998; Piskonen & Itävaara, 2004). A recent study in our laboratory has shown that spilled oil could be removed physically by gelatinization, and that bacteria naturally inhabiting the produced gels could biodegrade the oil (Radwan, Al‐Mailem, & Kansour, 2017). Unlike bioremediation, physical and chemical means are neither cost‐effective nor always eco‐friendly (Rosenberg, 1993).

Bioremediation is now accepted worldwide as the most promising technology for oil‐removal. It involves two distinct approaches, bioaugmentation and biostimulation (Atlas & Bartha, 1998). In bioaugmentation, the contaminated environments are inoculated exogenously with hydrocarbonoclastic microorganisms (Kuiper et al., 2004; Szulc et al., 2014; Van Limbergen, Top, & Verstraete, 1998). Such microorganisms are characterized by their ability to utilize crude oil or its constituents as sole sources of carbon and energy. Biostimulation, on the other hand, involves managements that enhance the indigenous (autochthonous), hydrocarbonoclastic microorganisms. This may be achieved by adding surfactants (Lawniczak, Marecik, & Chrzanowski, 2013; Szulc et al., 2014) and nitrogenous compounds and phosphates as microbial nutrients (Atlas & Bartha, 1998). For effective oil‐removal, bioaugmentation and biostimulation may be combined. Of course, bioaugmented microorganisms should first succeed in colonizing the contaminated environment (Radwan, 1991; Radwan, Sorkhoh, El‐Nemr, & El‐Desouky, 1997). In such a case, the practice is termed “autochthonous bioaugmentation” (ABA) (DiGregorio, Castglione, Gentini, & Lorenzi, 2015; Nikolopoulou, Pasadakis, & Kalogerakis, 2013; Ueno, Ito, Yumoto, & Okuyama, 2007).

The major objective of this study is to complement a recent work (Ali et al., 2016), in which we investigated the feasibility of autochthonous bioaugmentation for bench‐scale bioremediation of oil‐contaminated samples. However, in that study, a culture‐dependent method was used to analyze hydrocarbonoclastic microorganisms only using a culture‐dependent method. In the current paper, on the other hand, we analyzed the same environmental samples using the more comprehensive, molecular, culture‐independent approach. It is well known that culture‐dependent methods capture only a small portion of the microflora in the analyzed samples.

Within this context, nitrogenous compounds are essential for hydrocarbon‐biodegradation because nitrogen fertilization enhances oil‐bioremediation (Dashti, Ali, Eliyas et al., 2015; Klug & Markovetz, 1991; Leahy & Colwell, 1990; Radwan, 2008; Ratledge, 1978). Therefore, the total bacterial communities in the studied samples were surveyed for their constituent *nifH*‐genes which are known to code for nitrogenases. Diazotrophs (nitrogen‐fixing bacteria) carrying those genes in their genomes would potentially make the whole bacterial communities self‐sufficient in their nitrogen nutrition, which would be reflected in enhanced oil‐biodegradation.

## 2 EXPERIMENTAL PROCEDURES

### 2.1 Environmental materials for bioremediation and bioaugmentation

The materials to be bioremediated were seawater from the Arabian/Persian Gulf and desert soil from Kuwait. The materials were collected from sampling sites in the north, middle, and south of Kuwait (map in Figure S1). For bioaugmentation, pea (*Pisum sativum*) rhizosphere and a desert soil sample with 25‐year history of oil‐pollution were used. These two materials proved in earlier studies to be rich in hydrocarbonoclastic bacteria (Al‐Awadhi, Al‐Mailem, Dashti, Khanafer, & Radwan, 2012; Radwan, 2008). Intact plant roots with the adhering soil particles (rhizosphere) were detached, weighed, shaken (110 rpm) for 30 min in sterile water and the suspensions were used for bioaugmentation. Desert soil contaminated with oil since the famous 1991 spill associated with the second Gulf‐War were collected from Shuaybah (map), and used directly for bioaugmentation.

### 2.2 Experimental setup for bench‐scale bioremediation

Seawater aliquots, 50 ml, were distributed in 250 ml conical flasks and contaminated with 1%, w/v, portions of Kuwaiti light crude oil. Similarly, pristine (oil‐free) desert soil portions, 50 g, in conical flasks were provided with 50 ml aliquots of sterile water and contaminated with 1% portions of crude oil. One set of flasks (designated previously autoclaved) was sterilized by autoclaving and another set was left not autoclaved (designated fresh). The objective was to compare the behavior of the bioaugmented organisms in the presence and absence of the soil native microflora. The flask contents were bioaugmented with pea‐rhizosphere suspensions (volumes ≡ 2 g roots) or 2 g portions of the long‐polluted desert soil. The flasks were sealed and incubated at 30°C for 6 months. At time zero and periodically, replicate flasks were harvested for culture‐independent (molecular) analysis of their microbial communities and for quantitative analysis of the residual oil. This setup was the same used in the previous study (Ali et al., 2016), in which the culture‐dependent approach was adopted.

### 2.3 Oil‐consumption

At time zero and monthly, whole contents of flasks were extracted with three 20 ml successive aliquots of pentane. The volume of the combined extracts was completed to 60 ml, and 1 μl thereof was analyzed by gas liquid chromatography (GLC). For this, we used a Varian 3900 instrument (USA) equipped with an FID, a WCOT‐fused silica CP‐Sill‐5CB capillary column, and a program 45°C–310°C with temperature rising 10°C·min^−1^, and N_2_ as a carrier gas. The percentage of oil‐consumption was calculated as the percentage of total hydrocarbon peak areas based on the total areas of peaks in the GLC profiles at time zero.

### 2.4 Culture‐independent (molecular) analysis of bacterial communities

Total genomic DNA was extracted from the samples using a Rapid Water DNA Isolation Kit (MO‐BIO, Carlsbad, CA) and a Fast DNA Spin for Soil Kit (MP Biomedicals, LLC., France). The 16S rRNA‐genes in the extracts were amplified using the universal primer pair GM5F and 907R (Santegoeds, Ferdelman, Muyzer, & Beer, 1998). The resulting amplicons were resolved by DGGE using the Decode Universal Mutation Detection System (Bio‐Rad, California, USA). The acrylamide gels (45%–60%) were stained with SYBR Green, viewed with a Dark Reader transilluminator and gel bands were cut and stored in molecular water to elute the DNA. The 16S rDNAs were amplified, sequenced and the sequences were matched with GenBank database sequences of type strains.

### 2.5 Molecular analysis of *nifH*‐genes

The environmental samples were analyzed for nitrogen‐fixing bacterial communities through identifying the *nifH*‐genes, which are known to code for nitrogenase. The *nifH*‐genes in the total genomic DNA extracts were partially amplified using the primer combination PolFI (5′‐TGCGAICCSAAIGCIGACTC‐3′) and AQER‐GC30 (5′‐CGCCCGCCGCGCCCCGCGCCCGGCCCGCCCGACGATGTAGAT‐3′) (Wartiainen, Eriksson, Zheng, & Rasmussen, 2008). The amplification program consisted of initial denaturation at 95°C for 1 min, 35 cycles of denaturation at 95°C for 15 s, annealing at 55°C for 15 s and extension at 72°C for 30 s, and a final extension at 72°C for 7 min. The amplified genes were resolved by parallel DGGE with acrylamide gel concentrations of 45%–85%, sequenced and the sequences were deposited in the GenBank database under the accession numbers: KX611695–KX611787.

### 2.6 Statistical analysis

The oil‐consumption values were subjected to statistical analysis. Three replicates for each analysis were done and the mean values (±*SD* values) were calculated using Microsoft Excel 2007. The degree of significance was assessed using the Statistical Package for Social Sciences, version 12 and the analysis of variance (ANOVA) was used to differentiate between the means of the tested parameters.

## 3 RESULTS AND DISCUSSION

The Kuwait map (Figure S1) shows the sites from which the three seawater samples and the three desert soil samples were collected.

### 3.1 Oil‐consumption

**Figure 1:**
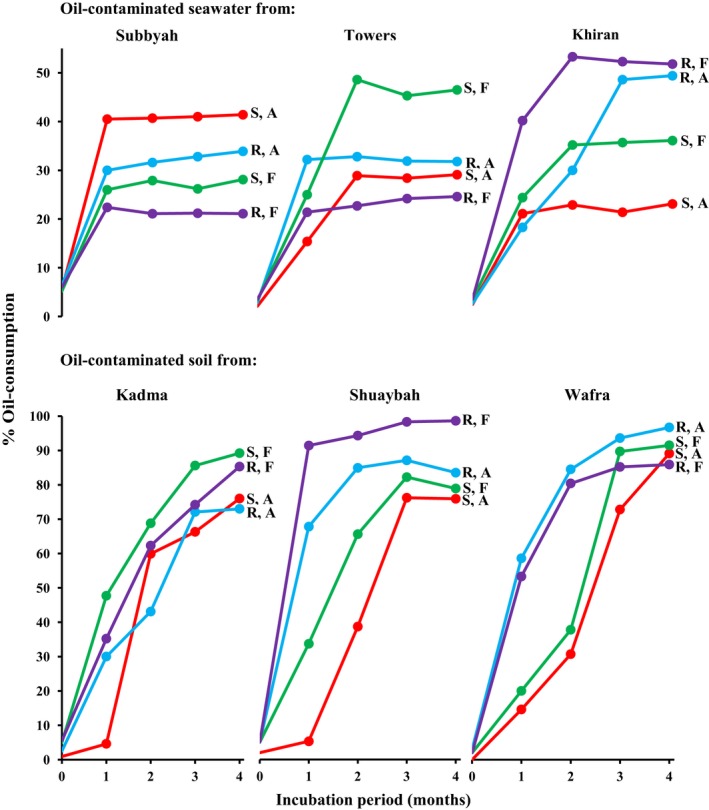
Oil‐consumption in seawater and desert soil batches bioaugmented with previously autoclaved (A) and with fresh (F) pea rhizosphere (R) and soil with long history of oil‐pollution (S)

Figure 1 shows that oil was removed in all of the seawater and desert soil batches studied. More oil was removed in the desert soil than in the seawater samples (*p *<* *.05). In soil samples, the maximum consumption values reached 98% (in the Shauybah and Wafra samples, whereas the maximum consumption values in the seawater samples did not exceed 53%). In many of the soil samples and some of the seawater samples, more oil was consumed in the fresh than in the previously autoclaved flasks (*p *<* *.05). Bioaugmentation using rhizospheric and soil microorganisms was thus, more effective in bioremediation of oily desert soil than oily seawater.

### 3.2 Dynamics of total bacterial communities during bioremediation of seawater samples

**Figure 2:**
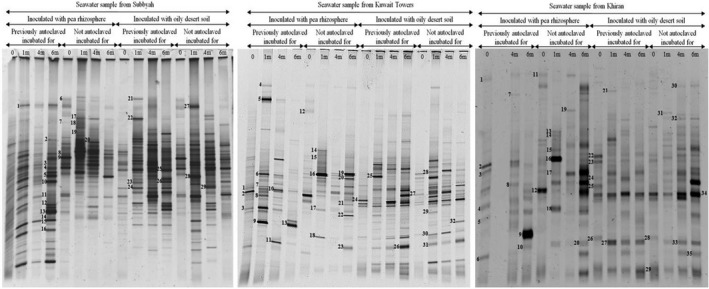
Typical DGGE gels of 16S rRNA‐genes amplified in total genomic DNA extracts from the three studied seawater samples. For band identification, see Figure 3 (see also Table S1)

**Figure 3:**
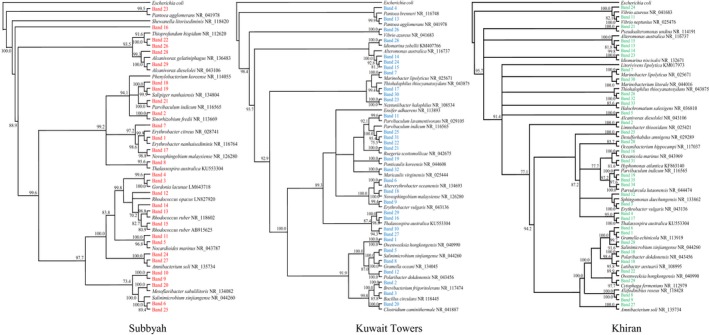
Phylogenetic trees based on the sequencing of the 16S rRNA‐gene bands in Figure 2 for the three studied seawater samples. Values shown in each node are the bootstrap values

The DGGE gels carrying the resolved 16S rRNA‐gene bands, that have been amplified in total genomic DNA extracts from the three studied seawater samples (Figure 2) revealed that the amplicon numbers in each sample were relatively high. However, only 29–35 bands on each gel were responded to the sequencing practice (Figure 3). The unsequenced bands (of the unidentified taxa) were even more in number than the sequenced ones. In the previous study on the same samples (Ali et al., 2016), the hydrocarbonoclastic species analyzed by the culture‐dependent technique (i.e., those capable of growth on oil or its constituents as sole sources of carbon and energy) did not exceed 7 species per seawater sample.

Figure 2 shows further that within each seawater sample, the amplicon numbers and band intensities varied during incubation for 6 months. This was true for samples bioaugmented with the pea rhizosphere and with the long‐contaminated desert soil. Such dynamic variations are probably due to dynamic changes of hydrocarbon intermediate products during various stages of bioremediation. Several bands were absent or faint at time zero but appeared or became denser at later stages. Such bands are apparently of those of the bacterial species which became involved in the relatively slow oil‐consumption in seawater (Figure 1). Examples of such bands were bands 1 (*Erythrobacter nanhaisediminis*), 8 (*Thalassospira australica*), 9 (*Phenylobacterium koreense*), and several others (see bands 3 through 16 and their identities in Figure 3) in seawater from Subbyah. For comparison, bioaugmented seawater samples analyzed by the culture‐dependent method revealed much fewer and completely different taxa viz. *Microbacterium oxidans*,* Rhodococcus* spp, in the rhizosphere‐bioaugmented batches and *Marinobacter hydrocarbonoclasticus* in the samples bioaugmented with the long‐contaminated soil (Ali et al., 2016). Other bands maintained nearly the same densities through the incubation period (e.g., band 2, *Sinorhizobium fredii* and several others) in the same seawater sample. Interestingly, the gene bands in the previously autoclaved seawater samples were more in number than in the fresh samples which had not been autoclaved. This may imply that the bioaugmented bacterial species suffered from competition with the native inhabitants of the (nonautoclaved) seawater samples, which evidently was reflected in the less effective oil‐removal rates than in the soil samples (see Figure 1).

The phylogenetic trees in Figure 3 were based on the results of sequencing of the 16S rRNA‐gene amplicons in Figure 2. More details are available in Table S1. As should be expected, the three seawater samples studied accommodated typical marine bacteria, for example, *Alcanivorax* spp., *Thalassospira* spp., *Marinobacter* spp., *Salinimicrobium* spp., *Alteromonas* spp., *Vibrio* spp. and several others. Particularly *Alcanivorax*,* Thalassospira*, and *Marinobacter* are even members of the so called obligate hydrocarbonoclastic bacteria (OHCB), a group considered as major hydrocarbon degraders in the marine ecosystem (Yakimov, Timmis, & Golyshin, 2007).

In addition to those autochthonous marine taxa, the bioaugmented seawater samples contained a few typical pea‐rhizospheric bacterial species (Ali et al., 2016) viz. *Rhodococcus* spp., *Ensifer* spp., and others. Many of the bacterial species in Figure 3 have also been reported in the literature as hydrocarbonoclastic and/or diazotrophic. Within this context, most of the hydrocarbonoclastic bacteria in the Gulf environment (probably elsewhere too) have diazotrophic potential (Dashti, Ali, Eliyas et al., 2015).

Although the three seawater samples were collected from the same water body, the Arabian/Persian Gulf, their bacterial communities were dramatically different. The sampling sites were about 50 km apart, which means that the physicochemical parameters prevailing in the three sites have been quite different.

### 3.3 Dynamics of total bacterial communities during bioremediation of soil samples

**Figure 4:**
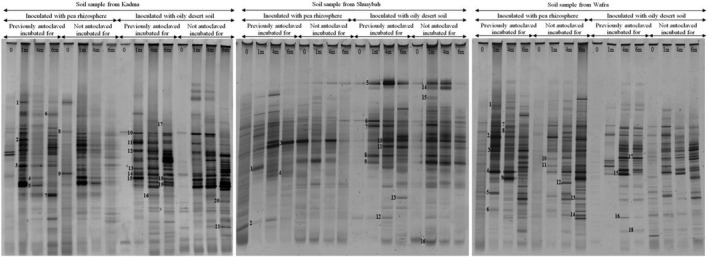
Typical DGGE gels of 16S rRNA‐genes amplified in total genomic DNA extracts from the three studied soil samples. For band identification, see Figure 5 (see also Table S2)

**Figure 5:**
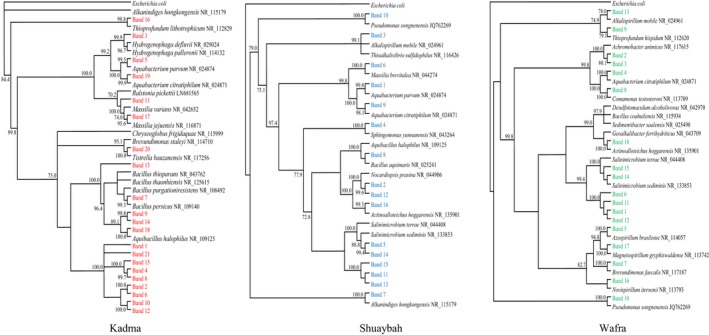
Phylogenetic trees based on the sequencing of the 16S rRNA‐gene bands in Figure 4 for the three studied soil samples. Values shown in each node are the bootstrap values

The DGGE profiles of 16S rRNA‐gene amplicons in total genomic DNA extracted from the three studied soil samples bioaugmented with pea rhizosphere and with the long‐polluted soil are presented in Figure 4. Like with the seawater samples, each soil sample exhibited relatively large numbers of amplicons with different intensities. However, the band numbers in the previously autoclaved samples were nearly similar to those in the nonautoclaved (fresh) samples. Differences were mainly in the band intensities. This similarity in band numbers suggests that the bioaugmented soil and rhizospheric bacteria lived in harmony with the native bacterial strains, and that they contributed together to the more effective oil‐degradation in the soil than the seawater batches (see Figure 1). Also here, only some bands ranging in number between 16 and 21 responded to the sequencing process (Figure 5). In the previous, culture‐dependent study on the same samples (Ali et al., 2016), the cultivable, hydrocarbonoclastic bacterial species in each sample were much lower in number. Commonly, the faintest amplicon bands were those in the time‐zero samples. Many bands, probably those of bacterial taxa particularly effective in oil‐degradation became denser with time. Examples are *Aquibacillus halophilus* (bands 4, 14, 18) *Aquabacterium* spp. (bands 5, 19), and *Bacillus* spp. (bands 7, 13, 15) in the Kadma soil; *Salinimicrobium sediminis* (bands 5, 14), *Aquibacillus halophilus*, (band 8), *Aquabacterium citratiphilum* (band 9), *Pseudomonas songnenensis* (band 10), and *Bacillus aquimaris* (band 11) in the Shuaybah soil; and *Achromobacter animicus* (bands 2, 3), *Aquabacterium citratiphilum* (band 4), *Brevundimonas faecalis* (band 7), *Comamonas testosterone* (band 8), *Bacillus aquimaris* (band 11), *Alkalispirillum mobile* (band 13), and *Salinimicrobium terrae* (band 15) in the Wafra soil. Other bands exhibited highest densities in certain months only, thus probably reflecting effectiveness in the utilization of transitional degradation intermediates of oil constituents.

Figure 5 shows the phylogenteic relationships among bacterial species in the studied soil samples (see also Table S2). The bacterial communities in the three soil samples were quite different. Only two genera, *Aquabacterium* and *Bacillus* were common in the three samples. *Brevundimonas* spp., *Aquibacillus halophilus*,* Alkanindiges hongkongensis*,* Massilia* spp., *Salinimicrobium sediminis* (and other species), *Pseudomonas songnenensis*,* Alkalispirillum mobile*, and *Actinoalloteichus hoggarensis* occurred in only two of the three soil samples. Otherwise, the bacterial taxa in the individual soil samples were limited to those samples only. As already mentioned, those differences are obviously correlated with differences in physicochemical parameters in the three samples. The dynamics of those bacterial species during bioremediation as illustrated in Figure 4, reflect the changes in the types of biodegradation intermediates during bioremediation. Interestingly, the bioaugmented soil samples harbored typical inhabitants of the long‐contaminated soil, for example, *Brevundimonas* spp., *Massilia* spp., *Pseudomonas* spp., and others. Based on that, bioaugmentation of freshly oil‐contaminated soil with long‐contaminated soil may merit the designation “autochthonous bioaugmentation.”

Many of the bacterial taxa in Figure 5 have been recognized earlier as hydrocarbonoclastic. These include the genera *Brevundimonas*,* Bacillus*,* Sphingomonas*, and *Pseudomonas* (Al‐Awadhi, Dashti, Kansour, Sorkhoh, & Radwan, 2012; Al‐Awadhi, Al‐Mailem et al., 2012). Several genera viz. *Bacillus*,* Nocardiopsis*,* Pseudomonas*,* Azospirillum*, and others are well known for their diazotrophic activity. These facts highlight the high oil‐bioremediation potential of the total bacterial communities in the bioaugmented soil samples, as analyzed by the culture‐independent approach. This is also consolidated by the oil‐consumption values in Figure 1, where no nitrogen fertilization was done during bioremediation.

### 3.4 Microbial response to oil in seawater and soil samples

The results in Figures 2, 3, 4, 5 were analyzed to define which microorganisms in seawater and soil were absent at time zero but appeared during the course of oil‐degradation. Of special interest was to learn whether some of such organisms were the same across the sites studied.

In the seawater sample from Subbyah, 9 species belonging to the genera, *Erythrobacter*,* Novosphingobium*,* Salipiger*,* Mesoflavibacter*,* Parvibaculum*,* Thioprofundum*,* Pantoea*,* Alcanivorax*, and *Amnibacterium* were recorded. In the seawater sample from Kuwait Towers, eight species belonging to the genera, *Pantoea*,* Marinobacter*,* Altererythrobacter*,* Salinimicrobium*,* Parvibaculum*,* Erythrobacter*,* Thalassospira*, and *Vibrio* were found. In the seawater sample from Khiran, nine species belonging to the genera, *Marinobacter*,* Aliifodinibius*,* Alteromonas*,* Oceanicola*,* Thalassospira*,* Lutibacter*,* Amnibacterium*,* Hyphomonas*, and *Parvibaculum* occurred. It is quite apparent that the taxa which inhabited the three sites simultaneously were few in number. *Parvibaculum* was the only representative.

In the soil sample from Kadma, species belonging to the five genera, *Aquibacillus*,* Thioprofundum*,* Aquabacterium*,* Bacillus*,* Massilia*,* Bacillus* and *Aquabacterium* were recorded. In the Shuaybah soil sample, there were 10 species belonging to the genera, *Alkalispirillum*,* Sphingomonas*,* Massilia*,* Alkanindiges*,* Bacillus*,* Aquabacterium*,* Pseudomonas*,* Nocardiopsis*,* Salinimicrobium*, and *Actinoalloteichus*. In the Wafra soil sample, species belonging to the 11 genera, *Salinimicrobium*,* Achromobacter*,* Aquabacterium*,* Brevundimonas*,* Comamonas*,* Thioprofundum*,* Pseudomonas*,* Alkalispirillum*,* Novispirillum*,* Magnetospirillum*, and *Actinoalloteichus* were identified. Obviously, the same taxa that responded to oil in the soil samples were more in number than the corresponding taxa in the seawater samples. Presumably, the “mixing effect” in the sea environment may be responsible for this interesting difference. This analysis also demonstrated the diversity of the microbial taxa in the open environment that respond to oil spills.

### 3.5 *nifH*‐Genes in total genomic DNA from seawater samples

**Figure 6:**
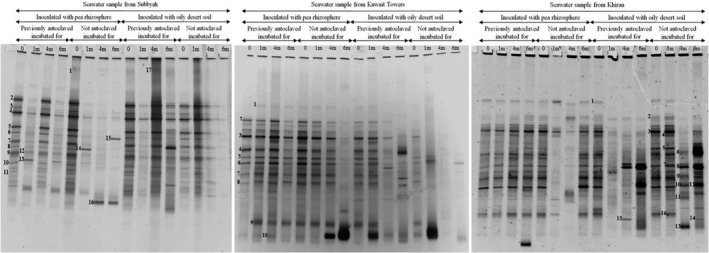
Typical DGGE gels of *nifH*‐genes amplified in total genomic DNA extracts from the three studied seawater samples. For band identification, see Figure 7 (see also Table S3)

Desert soil and seawater are naturally poor in nitrogenous nutrients. Therefore, diazotrophic (nitrogen‐fixing) microorganisms are potentially excellent natural sources of nitrogenous compounds in such ecosystems. With these facts in mind, the studied seawater and soil samples were analyzed for the *nifH*‐genes known to code for nitrogenase synthesis. Figure 6 shows typical DGGE profiles of *nifH*‐gene amplicons in the total DNA extracted from the three studied seawater samples.

Each sample exhibited several amplicon bands ranging in number between 10 and 16. Even at time zero, the amplicon bands were numerous and dense. During bioremediation, many bands persisted throughout, whereas others became weaker or disappeared with time, apparently because the denser genes were more effectively operating.

**Figure 7:**
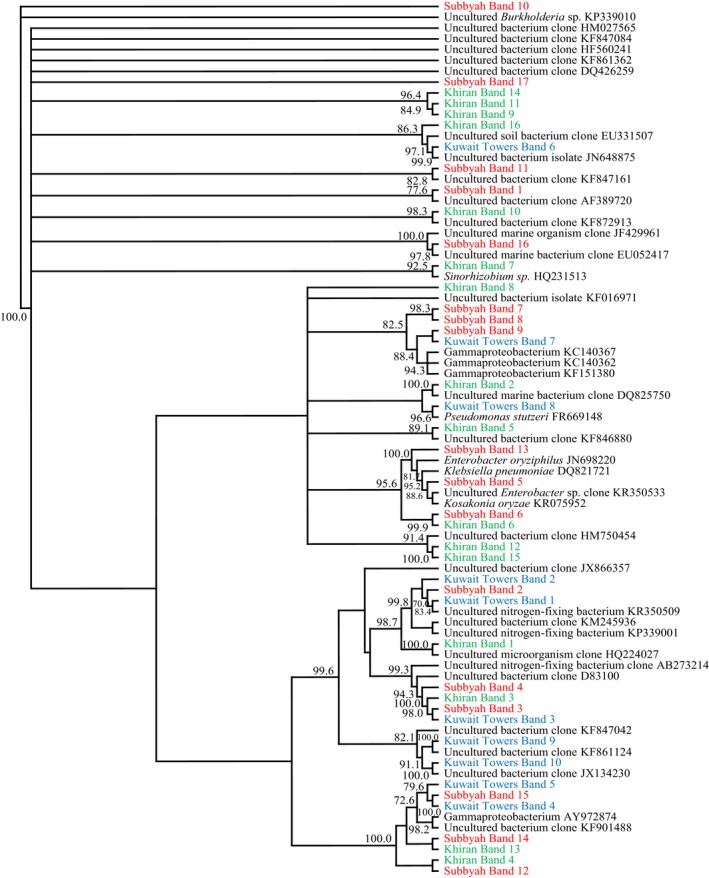
Phylogenetic trees based on the sequencing of the *nifH*‐gene bands in Figure 6 for the three studied seawater samples. Values shown in each node are the bootstrap values

The band sequencing in revealed that many of the amplicons in the three water samples were those of unidentified/uncultured bacterial taxa (Figure 7, see also Table S3). The high relatively numbers of such taxa suggests a rather high potential role in enriching the studied environments with nitrogenous nutrients. Figure 7 comprises several bacterial taxa known for their diazotrophic potential, for example, *Kosakonia oryzae* (Meng et al., 2015), *Burkholderia* spp. (Perin et al., 2006), *Klebsiella pneumoniae* (Al‐Awadhi, Al‐Mailem et al., 2012), *Enterobacter oryziphilus* (Hardoim et al., 2013), *Sinorhizobium* spp. (Haukka, Lindström, & Young, 1998), and others.

### 3.6 *nifH*‐Genes in total genomic DNA from soil samples

**Figure 8:**
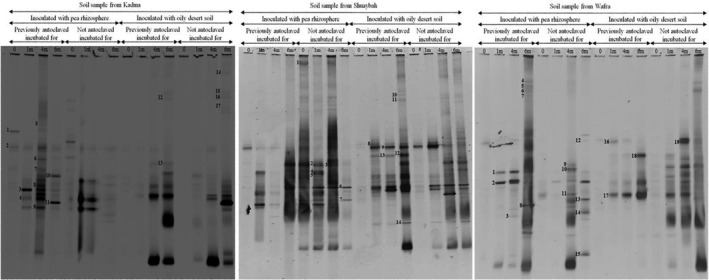
Typical DGGE gels of *nifH* ‐genes amplified in total genomic DNA extracts from the three studied soil samples. For band identification, see Figure 9 (see also Table S4)

**Figure 9:**
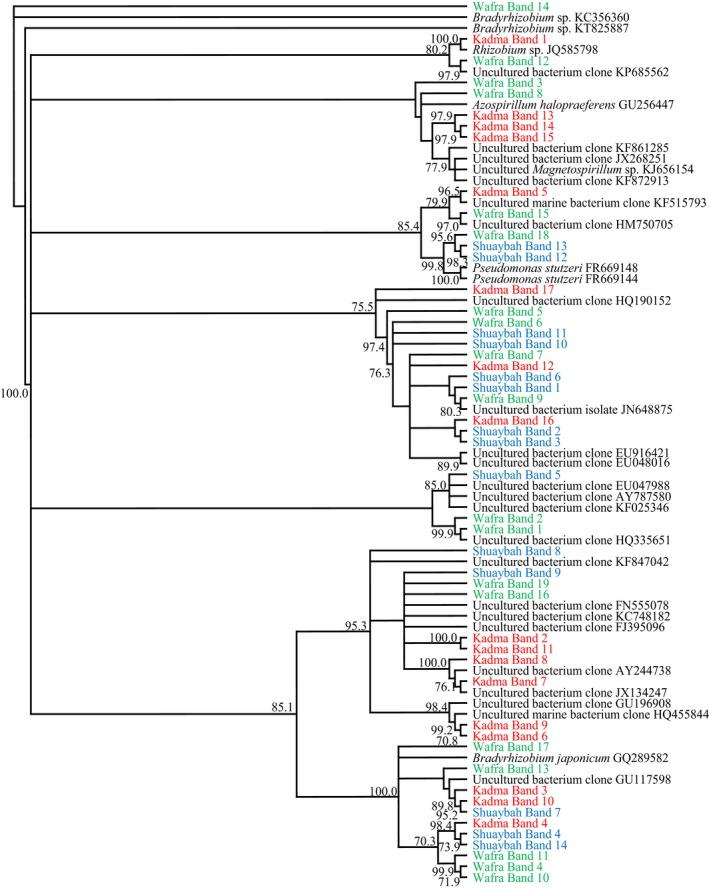
Phylogenetic trees based on the sequencing of the *nifH*‐gene bands in Figure 8 for the three studied soil samples. Values shown in each node are the bootstrap values

The DGGE gels of *nifH*‐genes in the three studied soil samples are illustrated in Figure 8, and the identities of the sequenced amplicons are listed in Figure 9.

The *nifH*‐gene band numbers successfully sequenced ranged in the three soil samples between 14 and 19 amplicons, that is, somewhat more than the numbers in the seawater samples. Figure 9 (see also Table S4) comprises, in addition to the predominant, unidentified/uncultured taxa, bacteria well known for their diazotrophic potential, for example, *Rhizobium* spp. (Dashti, Ali, Khanafer et al., 2015), *Pseudomonas stutzeri* (Al‐Awadhi, Al‐Mailem et al., 2012), *Azohydromonas lata* (Coelho et al., 2008), *Azospirillum rugosum* (Shime‐Hattori et al., 2011), *Bradyrhizobium japonicum* (Hennecke, 1990), and others. The results of the *nifH*‐gene analysis suggest that the microbial communities in the studied environmental samples should have been potentially self‐sufficient in their nitrogen nutrition. This is a prerequisite for effective oil‐biodegradation by hydrocarbonoclastic taxa without the use of exogenous nitrogenous fertilizers. It is true that the presence of the *nifH*‐genes alone does not indicate actual nitrogen‐fixation activity, yet it certainly reflects the nitrogen‐fixation potential.

## 4 CONCLUSIONS

The culture‐dependent approach used in the previous study for analysis of the same environmental samples provided the advantage of capturing selectively hydrocarbonoclastic bacteria. However, as already mentioned, a serious limitation of that approach is that it captures only a small portion of the total bacteria. The results of this study show that the culture‐independent approach revealed much more diverse hydrocarbonoclastic and diazotrophic bacterial taxa in the same environmental samples than those captured by the culture‐dependent method. The results confirmed that the bioaugmentation of oil‐contaminated desert soil with rhizospheric microflora or microflora in soil with long history of oil‐contamination brings about effective oil‐removal. On the other hand, using the same materials for the bioaugmentation of oily seawater is not as effective in oil‐removal. Presumably, desert soil and rhizospheric bacteria are better adopted to low salinity conditions. Therefore, their inoculation into seawater with about 3.5% NaCl concentration likely affected their survival. The importance of considering the source of material for bioaugmentation in relation to the contaminated habitat is a main conclusion of this study.

## CONFLICT OF INTEREST

The authors have no conflict of interest to declare.
