# Supplementary material for: Culture‐independent analysis of hydrocarbonoclastic bacterial communities in environmental samples during oil‐bioremediation
Source: Microbiologyopen. 2018 Apr 15;8(2):e00630. doi: 10.1002/mbo3.630 (PMC6391274; doi:10.1002/mbo3.630)
Supplement: Supplementary file 1 [file MBO3-8-e00630-s001.pdf]

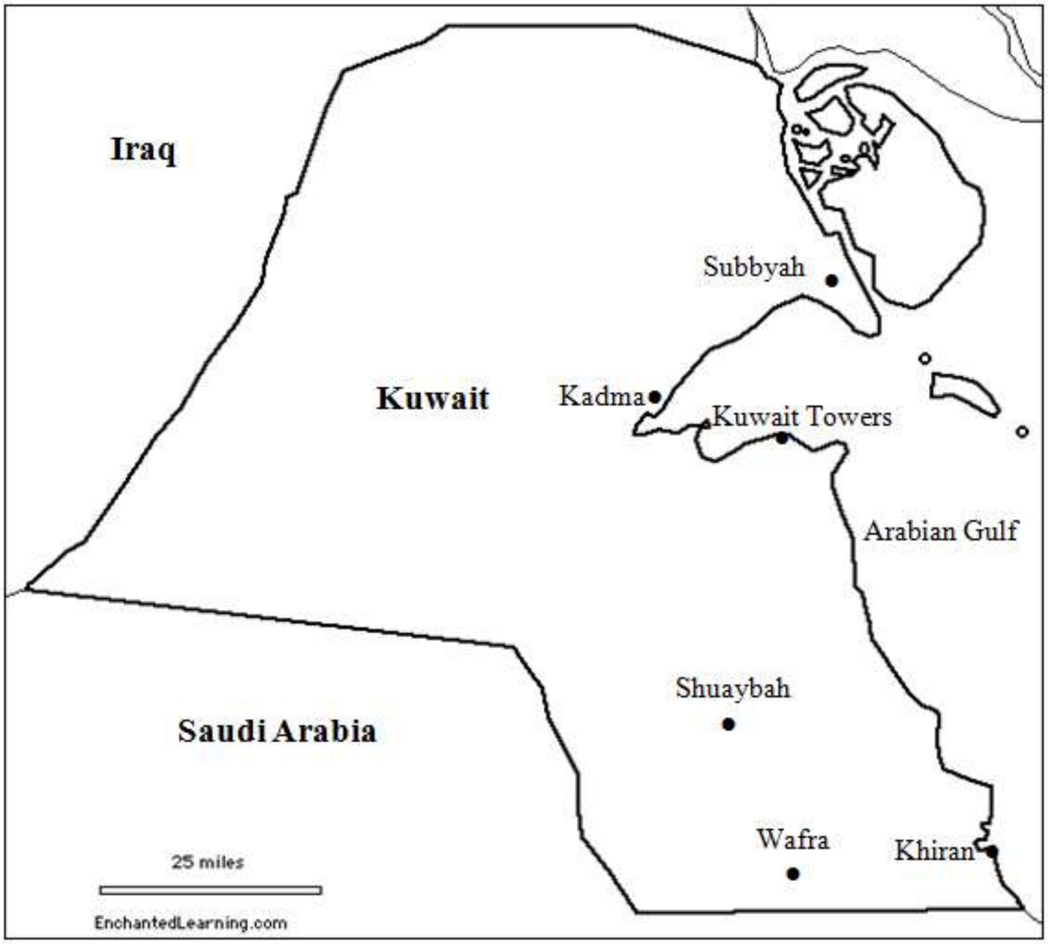

Figure S1 Kuwait map showing the sampling sites from which seawater and desert soil samples have been taken
