# Supplementary material for: Culture‐independent analysis of hydrocarbonoclastic bacterial communities in environmental samples during oil‐bioremediation
Source: Microbiologyopen. 2018 Apr 15;8(2):e00630. doi: 10.1002/mbo3.630 (PMC6391274; doi:10.1002/mbo3.630)
Supplement: Supplementary file 2 [file MBO3-8-e00630-s002.docx]

**TABLE S2** The results of sequencing of 16S rRNA-gene bands of the soil samples in Figure 3

| Band No. | Total bases | Nearest Gene Bank match (class, accession no.) | % Similarity |
| --- | --- | --- | --- |
| Soil sample from Kadma | | | |
| 1 | 425 | *Brevundimonas staleyi* (α-P, [NR_114710](https://www.ncbi.nlm.nih.gov/nucleotide/636558653?report=genbank&log$=nuclalign&blast_rank=1&RID=36A6G352014)) | 94 |
| 2 | 468 | *Hydrogenophaga defluvii* (β-P, [NR_029024](https://www.ncbi.nlm.nih.gov/nucleotide/265678719?report=genbank&log$=nuclalign&blast_rank=1&RID=36A6G352014)) | 97 |
| 3 | 478 | *Hydrogenophaga palleronii* (β-P, [NR_114132](https://www.ncbi.nlm.nih.gov/nucleotide/631252934?report=genbank&log$=nuclalign&blast_rank=1&RID=36A6G352014)) | 97 |
| 4 | 424 | *Aquibacillus halophilus* (Bac, [NR_109125](https://www.ncbi.nlm.nih.gov/nucleotide/961554979?report=genbank&log$=nuclalign&blast_rank=1&RID=36A6G352014)) | 95 |
| 5 | 519 | *Aquabacterium parvum* (β-P, [NR_024874](https://www.ncbi.nlm.nih.gov/nucleotide/219857244?report=genbank&log$=nuclalign&blast_rank=1&RID=36A6G352014)) | 99 |
| 6 | 412 | *Ralstonia pickettii* (β-P, [LN681565](https://www.ncbi.nlm.nih.gov/nucleotide/732170057?report=genbank&log$=nuclalign&blast_rank=1&RID=36A6G352014)) | 94 |
| 7 | 506 | *Bacillus persicus* (Bac, [NR_109140](https://www.ncbi.nlm.nih.gov/nucleotide/566085275?report=genbank&log$=nuclalign&blast_rank=1&RID=36A6G352014)) | 100 |
| 8 | 438 | *Aquibacillus halophilus* (Bac, [NR_109125](https://www.ncbi.nlm.nih.gov/nucleotide/961554979?report=genbank&log$=nuclalign&blast_rank=1&RID=36A6G352014)) | 97 |
| 9 | 437 | *Bacillus thaonhiensis* (Bac, [NR_125615](https://www.ncbi.nlm.nih.gov/nucleotide/672239026?report=genbank&log$=nuclalign&blast_rank=1&RID=36A6G352014)) | 97 |
| 10 | 471 | *Alkanindiges hongkongensis* (γ-P, [NR_115179](https://www.ncbi.nlm.nih.gov/nucleotide/636559122?report=genbank&log$=nuclalign&blast_rank=1&RID=36A6G352014)) | 96 |
| 11 | 501 | *Massilia varians* (β-P, NR_042652) | 98 |
| 12 | 471 | *Alkanindiges hongkongensis* (γ-P, [NR_115179](https://www.ncbi.nlm.nih.gov/nucleotide/636559122?report=genbank&log$=nuclalign&blast_rank=1&RID=36A6G352014)) | 98 |
| 13 | 410 | *Bacillus purgationiresistens* (Bac, [NR_108492](https://www.ncbi.nlm.nih.gov/nucleotide/566084949?report=genbank&log$=nuclalign&blast_rank=1&RID=36A6G352014)) | 93 |
| 14 | 499 | *Aquibacillus halophilus* (Bac, [NR_109125](https://www.ncbi.nlm.nih.gov/nucleotide/961554979?report=genbank&log$=nuclalign&blast_rank=1&RID=36A6G352014)) | 97 |
| 15 | 445 | *Bacillus thioparans* (Bac, NR_043762) | 94 |
| 16 | 442 | *Thioprofundum lithotrophicum* (γ-P, [NR_112829](https://www.ncbi.nlm.nih.gov/nucleotide/631251631?report=genbank&log$=nuclalign&blast_rank=1&RID=36A6G352014)) | 93 |
| 17 | 493 | *Massilia jejuensis* (β-P, [NR_116871](https://www.ncbi.nlm.nih.gov/nucleotide/636560811?report=genbank&log$=nuclalign&blast_rank=1&RID=36A6G352014)) | 99 |
| 18 | 518 | *Aquibacillus halophilus* (Bac, [NR_109125](https://www.ncbi.nlm.nih.gov/nucleotide/961554979?report=genbank&log$=nuclalign&blast_rank=1&RID=36A6G352014)) | 100 |
| 19 | 511 | *Aquabacterium citratiphilum* (β-P, [NR_024871](https://www.ncbi.nlm.nih.gov/nucleotide/219857243?report=genbank&log$=nuclalign&blast_rank=1&RID=36A6G352014)) | 99 |
| 20 | 461 | *Tistrella bauzanensis* (α-P, [NR_117256](https://www.ncbi.nlm.nih.gov/nucleotide/645319925?report=genbank&log$=nuclalign&blast_rank=1&RID=36A6G352014)) | 96 |
| 21 | 462 | *Chryseoglobus frigidaquae* (Act, [NR_115999](https://www.ncbi.nlm.nih.gov/nucleotide/636559939?report=genbank&log$=nuclalign&blast_rank=1&RID=36A6G352014)) | 96 |
| Soil sample from Shuaybah | | | |
| 1 | 537 | *Aquabacterium parvum* (β-P, [NR_024874](https://www.ncbi.nlm.nih.gov/nucleotide/219857244?report=genbank&log$=nuclalign&blast_rank=1&RID=36A6G352014)) | 99 |
| 2 | 438 | *Nocardiopsis prasina* (Act, [NR_044906](https://www.ncbi.nlm.nih.gov/nucleotide/343206314?report=genbank&log$=nuclalign&blast_rank=1&RID=36CBFU34014)) | 94 |
| 3 | 352 | *Thioalkalivibrio sulfidophilus* (γ-P, [NR_116426](https://www.ncbi.nlm.nih.gov/nucleotide/636560366?report=genbank&log$=nuclalign&blast_rank=3&RID=36CBFU34014)) | 90 |
| 4 | 373 | *Sphingomonas yunnanensis* (α-P, [NR_043264](https://www.ncbi.nlm.nih.gov/nucleotide/343202793?report=genbank&log$=nuclalign&blast_rank=3&RID=36CBFU34014)) | 91 |
| 5 | 467 | *Salinimicrobium sediminis* (Fla, [NR_133853](https://www.ncbi.nlm.nih.gov/nucleotide/959495001?report=genbank&log$=nuclalign&blast_rank=1&RID=36CBFU34014)) | 96 |
| 6 | 517 | *Massilia brevitalea* (β-P, [NR_044274](https://www.ncbi.nlm.nih.gov/nucleotide/343198997?report=genbank&log$=nuclalign&blast_rank=1&RID=36CBFU34014)) | 99 |
| 7 | 456 | *Alkanindiges hongkongensis* (γ-P, [NR_115179](https://www.ncbi.nlm.nih.gov/nucleotide/636559122?report=genbank&log$=nuclalign&blast_rank=1&RID=36A6G352014)) | 95 |
| 8 | 443 | *Aquibacillus halophilus* (Bac, [NR_109125](https://www.ncbi.nlm.nih.gov/nucleotide/961554979?report=genbank&log$=nuclalign&blast_rank=1&RID=36A6G352014)) | 98 |
| 9 | 454 | *Aquabacterium citratiphilum* (β-P, [NR_024871](https://www.ncbi.nlm.nih.gov/nucleotide/219857243?report=genbank&log$=nuclalign&blast_rank=1&RID=36A6G352014)) | 96 |
| 10 | 446 | *Pseudomonas songnenensis* (γ-P, [JQ762269](https://www.ncbi.nlm.nih.gov/nucleotide/720105534?report=genbank&log$=nuclalign&blast_rank=1&RID=36CBFU34014)) | 98 |
| 11 | 392 | *Bacillus aquimaris* (Bac, [NR_025241](https://www.ncbi.nlm.nih.gov/nucleotide/219857652?report=genbank&log$=nuclalign&blast_rank=1&RID=36CBFU34014)) | 94 |
| 12 | 441 | *Nocardiopsis prasina* (Act, [NR_044906](https://www.ncbi.nlm.nih.gov/nucleotide/343206314?report=genbank&log$=nuclalign&blast_rank=1&RID=36CBFU34014)) | 93 |
| 13 | 419 | *Alkalispirillum mobile* (γ-P, [NR_024961](https://www.ncbi.nlm.nih.gov/nucleotide/219857373?report=genbank&log$=nuclalign&blast_rank=1&RID=36CBFU34014)) | 92 |
| 14 | 478 | *Salinimicrobium sediminis* (Fla, [NR_133853](https://www.ncbi.nlm.nih.gov/nucleotide/959495001?report=genbank&log$=nuclalign&blast_rank=1&RID=36CBFU34014)) | 98 |
| 15 | 442 | *Salinimicrobium terrae* (Fla, [NR_044408](https://www.ncbi.nlm.nih.gov/nucleotide/343205911?report=genbank&log$=nuclalign&blast_rank=1&RID=36CBFU34014)) | 94 |
| 16 | 450 | *Actinoalloteichus hoggarensis* (Act, [NR_135901](https://www.ncbi.nlm.nih.gov/nucleotide/1013172434?report=genbank&log$=nuclalign&blast_rank=2&RID=36CBFU34014)) | 95 |
| Soil sample from Wafra | | | |
| 1 | 374 | *Sedimentibacter saalensis* (Bac, [NR_025498](https://www.ncbi.nlm.nih.gov/nucleotide/219878359?report=genbank&log$=nuclalign&blast_rank=1&RID=36DBKR8P015)) | 91 |
| 2 | 522 | *Achromobacter animicus* (β-P, [NR_117615](https://www.ncbi.nlm.nih.gov/nucleotide/645320401?report=genbank&log$=nuclalign&blast_rank=1&RID=36DBKR8P015)) | 99 |
| 3 | 497 | *Achromobacter animicus* (β-P, [NR_117615](https://www.ncbi.nlm.nih.gov/nucleotide/645320401?report=genbank&log$=nuclalign&blast_rank=1&RID=36DBKR8P015)) | 99 |
| 4 | 391 | *Aquabacterium citratiphilum* (β-P, [NR_024871](https://www.ncbi.nlm.nih.gov/nucleotide/219857243?report=genbank&log$=nuclalign&blast_rank=1&RID=36A6G352014)) | 100 |
| 5 | 504 | *Azospirillum brasilense* (γ-P, [NR_114057](https://www.ncbi.nlm.nih.gov/nucleotide/631252859?report=genbank&log$=nuclalign&blast_rank=5&RID=36DBKR8P015)) | 99 |
| 6 | 258 | *Geoalkalibacter ferrihydriticus* (α-P, [NR_043709](https://www.ncbi.nlm.nih.gov/nucleotide/343198800?report=genbank&log$=nuclalign&blast_rank=10&RID=36DBKR8P015)) | 84 |
| 7 | 431 | *Brevundimonas faecalis* (α-P, [NR_117187](https://www.ncbi.nlm.nih.gov/nucleotide/645319831?report=genbank&log$=nuclalign&blast_rank=1&RID=36DBKR8P015)) | 94 |
| 8 | 513 | *Comamonas testosteroni* (β-P, [NR_113709](https://www.ncbi.nlm.nih.gov/nucleotide/631252511?report=genbank&log$=nuclalign&blast_rank=1&RID=36DBKR8P015)) | 99 |
| 9 | 354 | *Thioprofundum hispidum* (γ-P, [NR_112620](https://www.ncbi.nlm.nih.gov/nucleotide/631251423?report=genbank&log$=nuclalign&blast_rank=1&RID=36DBKR8P015)) | 93 |
| 10 | 510 | *Pseudomonas songnenensis* (γ-P, [JQ762269](https://www.ncbi.nlm.nih.gov/nucleotide/720105534?report=genbank&log$=nuclalign&blast_rank=1&RID=36CBFU34014)) | 98 |
| 11 | 372 | *Bacillus coahuilensis* (Bac, [NR_115934](https://www.ncbi.nlm.nih.gov/nucleotide/636559874?report=genbank&log$=nuclalign&blast_rank=1&RID=36DBKR8P015)) | 92 |
| 12 | 427 | *Desulfotomaculum alcoholivorax* (Bac, [NR_042970](https://www.ncbi.nlm.nih.gov/nucleotide/343202594?report=genbank&log$=nuclalign&blast_rank=1&RID=36DBKR8P015)) | 95 |
| 13 | 401 | *Alkalispirillum mobile* (γ-P, [NR_024961](https://www.ncbi.nlm.nih.gov/nucleotide/219857373?report=genbank&log$=nuclalign&blast_rank=1&RID=36CBFU34014)) | 93 |
| 14 | 482 | *Salinimicrobium sediminis* (Fla, [NR_133853](https://www.ncbi.nlm.nih.gov/nucleotide/959495001?report=genbank&log$=nuclalign&blast_rank=1&RID=36CBFU34014)) | 98 |
| 15 | 527 | *Salinimicrobium terrae* (Fla, [NR_044408](https://www.ncbi.nlm.nih.gov/nucleotide/343205911?report=genbank&log$=nuclalign&blast_rank=1&RID=36CBFU34014)) | 99 |
| 16 | 353 | *Novispirillum itersonii* (α-P, [NR_113793](https://www.ncbi.nlm.nih.gov/nucleotide/343205911?report=genbank&log$=nuclalign&blast_rank=1&RID=36DBKR8P015)) | 89 |
| 17 | 379 | *Magnetospirillum gryphiswaldense* (α-P, [NR_113742](https://www.ncbi.nlm.nih.gov/nucleotide/631252544?report=genbank&log$=nuclalign&blast_rank=4&RID=36DBKR8P015)) | 97 |
| 18 | 458 | *Actinoalloteichus hoggarensis* (Act, [NR_135901](https://www.ncbi.nlm.nih.gov/nucleotide/1013172434?report=genbank&log$=nuclalign&blast_rank=2&RID=36CBFU34014)) | 95 |

α-P, α-Proteobacteria; β-P, β-Proteobacteria; γ-P, γ-Proteobacteria; Act, Actinobacteria; Fla, Flavobacteriia; Bac, Bacilli
