# Supplementary material for: Culture‐independent analysis of hydrocarbonoclastic bacterial communities in environmental samples during oil‐bioremediation
Source: Microbiologyopen. 2018 Apr 15;8(2):e00630. doi: 10.1002/mbo3.630 (PMC6391274; doi:10.1002/mbo3.630)
Supplement: Supplementary file 3 [file MBO3-8-e00630-s003.docx]

**TABLE S3** The results of sequencing of *nifH*-gene bands of the seawater samples in Figure 5

| Band No. | Total bases | Nearest Gene Bank match (class, accession no.) | % Similarity |
| --- | --- | --- | --- |
| Seawater sample from Subbyah | | | |
| **1** | 147 | Uncultured bacterium clone SIW1-2, (*nifH*) gene (AF389720) | **91** |
| **2** | 186 | Uncultured bacterium clone 10-2_C5, (*nifH*) gene (KM245936) | **90** |
| **3** | 120 | Uncultured nitrogen-fixing bacterium clone: Nd22, (*nifH*) gene (AB273214) | **85** |
| 4 | 159 | Uncultured bacterium clone: TKG7, (*nifH*) gene (D83100) | **86** |
| 5 | 216 | *Kosakonia oryzae* strain R5-424, (*nifH*) gene (γ-P, KR075952) | **99** |
| 6 | 162 | *Enterobacter oryziphilus* strain REICA_142, (*nifH*) gene (γ-P, JN698220) | **90** |
| 7 | 189 | Gammaproteobacterium BAL508, (*nifH*) gene (γ-P, KF151380) | **90** |
| 8 | 201 | Gammaproteobacterium BAL281, (*nifH*) gene (γ-P, AY972874) | **91** |
| 9 | 207 | Gammaproteobacterium BAL410 clone 663_1M, (*nifH*) gene (γ-P, KC140367) | **96** |
| 10 | 228 | Uncultured *Burkholderia* sp. isolate DGGE gel band NF_TMPC12, (*nifH*) gene (β-P, KP339010) | **99** |
| 11 | 168 | Uncultured bacterium clone MDE_elv_20d8, (*nifH*) gene (KF847161) | **95** |
| 12 | 123 | Uncultured bacterium clone F1_663M, (*nifH*) gene (JX866357) | **84** |
| 13 | 246 | *Klebsiella pneumoniae* nitrogenase iron protein, (*nifH*) gene (γ-P, DQ821721) | **95** |
| 14 | 195 | Uncultured marine organism clone S2D150M15, (*nifH*) gene (JF429961) | **95** |
| 15 | 216 | Uncultured *Enterobacter* sp. clone NF_TBGSS5, (*nifH*) gene (γ-P, KR350533) | **100** |
| 16 | 243 | Uncultured marine bacterium clone 24540A01, (*nifH*) gene (EU052417) | **99** |
| 17 | 222 | Uncultured bacterium clone 29.1 *NifH*-like (*nifH*) gene (DQ426259) | **93** |
| Seawater sample from Kuwait Towers | | | |
| 1 | 239 | Uncultured nitrogen-fixing bacterium isolate DGGE gel band NF_USTRW8, (*nifH*) gene (KR350509) | 97 |
| 2 | 219 | Uncultured nitrogen-fixing bacterium isolate DGGE gel band NF_TMPC3, (*nifH*) gene (KP339001) | 96 |
| 3 | 146 | Uncultured nitrogen-fixing bacterium clone: Nd22, (*nifH*) gene (AB273214) | 84 |
| 4 | 240 | *Kosakonia oryzae* strain R5-424, (*nifH*) gene (γ-P, KR075952) | 100 |
| 5 | 156 | *Enterobacter oryziphilus* strain REICA_142, (*nifH*) gene (γ-P, JN698220) | 88 |
| 6 | 255 | Uncultured bacterium isolate DGGE gel band 18r, (*nifH*) gene (JN648875) | 98 |
| 7 | 261 | Gamma proteobacterium BAL407 clone 365_K1, (*nifH*) gene (γ-P, KC140362) | 97 |
| 8 | 243 | *Pseudomonas stutzeri* strain Gr65, (*nifH*) gene (γ-P, FR669148) | 95 |
| 9 | 207 | Uncultured bacterium clone NA98, (*nifH*) gene (KF861124) | 93 |
| 10 | 234 | Uncultured bacterium clone Enif1_1, (*nifH*) gene (JX134230) | 94 |
| Seawater sample from Khiran | | | |
| 1 | 129 | Uncultured microorganism clone E407-63, (*nifH*) gene (HQ224027) | 87 |
| 2 | 228 | Uncultured marine bacterium clone RS_5674A4, (*nifH*) gene (DQ825750) | 97 |
| 3 | 135 | Uncultured nitrogen-fixing bacterium clone: Nd22, (*nifH*) gene (AB273214) | 84 |
| 4 | 117 | Uncultured bacterium clone MDE_elv_14g11, (*nifH*) gene (KF847042) | 86 |
| 5 | 153 | Uncultured bacterium clone MDE_amb_35e11, (*nifH*) gene (KF846880) | 88 |
| 6 | 147 | *Enterobacter oryziphilus* strain REICA_142, (*nifH*) gene (γ-P, JN698220) | 88 |
| 7 | 183 | *Sinorhizobium sp.* CCBAU 25426, (*nifH*) gene (α-P, HQ231513) | 93 |
| 8 | 192 | Uncultured bacterium isolate DGGE gel band n10-3-1, (*nifH*) gene (KF016971) | 89 |
| 9 | 129 | Uncultured bacterium clone MDE_elv_17c6, (*nifH*) gene (KF8470840) | 87 |
| 10 | 216 | Uncultured bacterium clone IFRpool-20 *NifH*, (*nifH*) gene (KF872913) | 92 |
| 11 | 189 | Uncultured bacterium clone pPS896, (*nifH*) gene (HM027565) | 92 |
| 12 | 216 | Uncultured bacterium clone BE326FW120712BH2_OTU5, (*nifH*) gene (KF901488) | 91 |
| 13 | 135 | Uncultured bacterium clone NC166, (*nifH*) gene (KF861362) | 88 |
| 14 | 155 | Uncultured bacterium clone 08822-24(MOTU1084), (*nifH*) gene (HF560241) | 93 |
| 15 | 171 | Uncultured bacterium clone JSS1-12, (*nifH*) gene (HM750454) | 93 |
| 16 | 180 | Uncultured soil bacterium clone SC16 *NifH*, (*nifH*) gene (EU331507) | 93 |

α-P, α-Proteobacteria; β-P, β-Proteobacteria; γ-P, γ-Proteobacteria
