# Supplementary material for: Culture‐independent analysis of hydrocarbonoclastic bacterial communities in environmental samples during oil‐bioremediation
Source: Microbiologyopen. 2018 Apr 15;8(2):e00630. doi: 10.1002/mbo3.630 (PMC6391274; doi:10.1002/mbo3.630)
Supplement: Supplementary file 4 [file MBO3-8-e00630-s004.docx]

**TABLE S4** The results of sequencing of *nifH*-gene bands of the soil samples in Figure 7

| Band No. | Total bases | Nearest Gene Bank match (class, accession no.) | % Similarity |  |
| --- | --- | --- | --- | --- |
| Soil sample from Kadma | | | | |
| 1 | 258 | *Rhizobium* sp. CCBAU 23250, (*nifH*) gene (α-P, JQ585798) | 100 | |
| 2 | 162 | Uncultured bacterium clone 9007-5, (*nifH*) gene (FJ395096) | 86 | |
| 3 | 201 | Uncultured *Magnetospirillum* sp. clone D22, (*nifH*) gene (KJ656154) | 97 | |
| 4 | 176 | Uncultured bacterium clone IPA46, (*nifH*) gene (EU047988) | 95 | |
| 5 | 204 | Uncultured marine bacterium clone ETSP_OMZ_44158A51, (*nifH*) gene (KF515793) | 91 | |
| 6 | 135 | Uncultured marine bacterium clone A9-0M-12, (*nifH*) gene (HQ455844) | 87 | |
| 7 | 213 | Uncultured bacterium clone Enif1_18, (*nifH*) gene (JX134247) | 96 | |
| 8 | 159 | Uncultured bacterium clone GN823A25, (*nifH*) gene (AY244738) | 92 | |
| 9 | 123 | Uncultured bacterium clone P322, (*nifH*) gene (GU196908) | 83 | |
| 10 | 186 | Uncultured bacterium clone NB182, (*nifH*) gene (KF861285) | 97 | |
| 11 | 162 | Uncultured bacterium clone 9007-5, (*nifH*) gene (FJ395096) | 86 | |
| 12 | 243 | Uncultured bacterium isolate DGGE gel band 18r, (*nifH*) gene (JN648875) | 97 | |
| 13 | 183 | Uncultured bacterium clone IFRpool-20, (*nifH*) gene (KF872913) | 90 | |
| 14 | 189 | *Azospirillum halopraeferens* strain DSM 3675, (*nifH*) gene (α-P, GU256447) | 92 | |
| 15 | 192 | Uncultured bacterium clone IFRpool-20, (*nifH*) gene (KF872913) | 91 | |
| 16 | 234 | Uncultured bacterium isolate DGGE gel band 18r, (*nifH*) gene (JN648875) | 97 | |
| 17 | 189 | Uncultured bacterium clone BRnif7, (*nifH*) gene (HQ190152) | 91 | |
| Soil sample from Shuaybah | | | | |
| 1 | 213 | Uncultured bacterium isolate DGGE gel band 18r, (*nifH*) gene (JN648875) | 98 | |
| 2 | 246 | Uncultured bacterium isolate DGGE gel band 18r, (*nifH*) gene (JN648875) | 97 | |
| 3 | 255 | Uncultured bacterium isolate DGGE gel band 18r, (*nifH*) gene (JN648875) | 96 | |
| 4 | 246 | Uncultured bacterium clone IPA46, (*nifH*) gene (EU047988) | 95 | |
| 5 | 198 | Uncultured bacterium clone 42_Z65C, (*nifH*) gene (AY787580) | 96 | |
| 6 | 255 | Uncultured bacterium isolate DGGE gel band 18r, (*nifH*) gene (JN648875) | 98 | |
| 7 | 222 | Uncultured bacterium clone IFRpool-20, (*nifH*) gene (KF872913) | 96 | |
| 8 | 153 | Uncultured bacterium clone MDE_elv_14g11, (*nifH*) gene (KF847042) | 96 | |
| 9 | 156 | Uncultured bacterium clone SYN102, (*nifH*) gene (KC748182) | 87 | |
| 10 | 240 | Uncultured bacterium isolate DGGE gel band 18r, (*nifH*) gene (JN648875) | 96 | |
| 11 | 255 | Uncultured bacterium isolate DGGE gel band 18r, (*nifH*) gene (JN648875) | 96 | |
| 12 | 246 | *Pseudomonas stutzeri* strain Gr65, (*nifH*) gene (γ-P, FR669148) | 95 | |
| 13 | 234 | *Pseudomonas stutzeri* strain Gr57, (*nifH*) gene (γ-P, FR669144) | 95 | |
| 14 | 240 | Uncultured bacterium clone HJS_Wat03, (*nifH*) gene (KF025346) | 95 | |
| Soil sample from Wafra | | | | |
| 1 | 252 | Uncultured bacterium clone KW-C6, (*nifH*) gene (HQ335651) | 99 | |
| 2 | 186 | Uncultured bacterium clone KW-C6, (*nifH*) gene (HQ335651) | 98 | |
| 3 | 192 | Uncultured bacterium clone PN25, (*nifH*) gene (GU117598) | 95 | |
| 4 | 195 | Uncultured bacterium isolate DGGE gel band 18r, (*nifH*) gene (JN648875) | 93 | |
| 5 | 237 | Uncultured bacterium isolate DGGE gel band 18r, (*nifH*) gene (JN648875) | 95 | |
| 6 | 201 | Uncultured bacterium clone bal2julF10, (*nifH*) gene (EU916421) | 96 | |
| 7 | 264 | Uncultured bacterium clone bal2julF10, (*nifH*) gene (EU916421) | 96 | |
| 8 | 183 | Uncultured bacterium clone cloA-15, (*nifH*) gene JX268251) | 95 | |
| 9 | 249 | Uncultured bacterium isolate DGGE gel band 18r, (*nifH*) gene (JN648875) | 99 | |
| 10 | 210 | Uncultured bacterium isolate DGGE gel band 18r, (*nifH*) gene (JN648875) | 97 | |
| 11 | 171 | Uncultured bacterium clone IPA74, (*nifH*) gene (EU048016) | 92 | |
| 12 | 153 | Uncultured bacterium clone nf12, (*nifH*) gene (KP685562) | 89 | |
| 13 | 204 | *Bradyrhizobium* sp. INPA01-384B, (*nifH*) gene (α-P, KT825887) | 96 | |
| 14 | 198 | *Bradyrhizobium* sp. CCBAU 101065, (*nifH*) gene (α-P, KC356360) | 99 | |
| 15 | 213 | Uncultured bacterium clone SVS10-5, (*nifH*) gene (HM750705) | 92 | |
| 16 | 147 | Uncultured bacterium clone DNA_F03, (*nifH*) gene (FN555078) | 89 | |
| 17 | 213 | Uncultured bacterium clone KW-C6, (*nifH*) gene (HQ335651) | 97 | |
| 18 | 207 | *Pseudomonas stutzeri* strain Gr65, (*nifH*) gene (γ-P, FR669148) | 97 | |
| 19 | 153 | *Bradyrhizobium japonicum* clone nifH2-70, (*nifH*) gene (α-P, GQ289582) | 89 | |

α-P, α-Proteobacteria; β-P, β-Proteobacteria; γ-P, γ-Proteobacteria.
