# Supplementary material for: Culture‐independent analysis of hydrocarbonoclastic bacterial communities in environmental samples during oil‐bioremediation
Source: Microbiologyopen. 2018 Apr 15;8(2):e00630. doi: 10.1002/mbo3.630 (PMC6391274; doi:10.1002/mbo3.630)
Supplement: Supplementary file 5 [file MBO3-8-e00630-s005.docx]

**TABLE S1** The results of sequencing of 16S rRNA-gene bands of the seawater samples in Figure 1

| Band No. | Total bases | Nearest Gene Bank match (class, accession no.) | % Similarity |
| --- | --- | --- | --- |
| Seawater sample from Subbyah | | | |
| 1 | 476 | *Erythrobacter nanhaisediminis* (α-P, [NR_116764](https://www.ncbi.nlm.nih.gov/nucleotide/636560704?report=genbank&log$=nuclalign&blast_rank=1&RID=36M1KNZR015)) | **99** |
| 2 | 453 | *Sinorhizobium fredii* (α-P, [NR_113669](https://www.ncbi.nlm.nih.gov/nucleotide/631252471?report=genbank&log$=nuclalign&blast_rank=3&RID=36M1KNZR015)) | **98** |
| 3 | 497 | *Gordonia lacunae* (Act, [LM643718](https://www.ncbi.nlm.nih.gov/nucleotide/669173707?report=genbank&log$=nuclalign&blast_rank=3&RID=36M1KNZR015)) | **99** |
| 4 | 488 | *Gordonia lacunae* (Act, [LM643718](https://www.ncbi.nlm.nih.gov/nucleotide/669173707?report=genbank&log$=nuclalign&blast_rank=3&RID=36M1KNZR015)) | **97** |
| 5 | 478 | *Nocardioides marinus* (Act, [NR_043787](https://www.ncbi.nlm.nih.gov/nucleotide/343204524?report=genbank&log$=nuclalign&blast_rank=1&RID=36M1KNZR015)) | **99** |
| 6 | 459 | *Salinimicrobium xinjiangense* (Fla, [NR_044260](https://www.ncbi.nlm.nih.gov/nucleotide/343198991?report=genbank&log$=nuclalign&blast_rank=1&RID=36E3JWYT015)) | **97** |
| 7 | 419 | *Erythrobacter citreus* (α-P, [NR_028741](https://www.ncbi.nlm.nih.gov/nucleotide/265678439?report=genbank&log$=nuclalign&blast_rank=1&RID=36M1KNZR015)) | **94** |
| 8 | 305 | *Thalassospira australica* (α-P, [KU553304](https://www.ncbi.nlm.nih.gov/nucleotide/983964128?report=genbank&log$=nuclalign&blast_rank=1&RID=36E3JWYT015)) | **88** |
| 9 | 483 | *Phenylobacterium koreense* (α-P, [NR_114055](https://www.ncbi.nlm.nih.gov/nucleotide/631252857?report=genbank&log$=nuclalign&blast_rank=2&RID=36M1KNZR015)) | **97** |
| 10 | 490 | *Rhodococcus opacus* (Act, [LN827920](https://www.ncbi.nlm.nih.gov/nucleotide/770465284?report=genbank&log$=nuclalign&blast_rank=1&RID=36M1KNZR015)) | **97** |
| 11 | 505 | *Nocardioides marinus* (Act, [NR_043787](https://www.ncbi.nlm.nih.gov/nucleotide/343204524?report=genbank&log$=nuclalign&blast_rank=1&RID=36M1KNZR015)) | **98** |
| 12 | 522 | *Rhodococcus opacus* (Act, [NR_043787](https://www.ncbi.nlm.nih.gov/nucleotide/343204524?report=genbank&log$=nuclalign&blast_rank=1&RID=36M1KNZR015)) | **99** |
| 13 | 493 | *Rhodococcus ruber* (Act, [NR_118602](https://www.ncbi.nlm.nih.gov/nucleotide/645321698?report=genbank&log$=nuclalign&blast_rank=1&RID=36M1KNZR015)) | **100** |
| 14 | 461 | *Rhodococcus gordoniae* (Act, [AB915625](https://www.ncbi.nlm.nih.gov/nucleotide/592971050?report=genbank&log$=nuclalign&blast_rank=1&RID=36M1KNZR015)) | **95** |
| 15 | 487 | *Rhodococcus gordoniae* (Act, [AB915625](https://www.ncbi.nlm.nih.gov/nucleotide/592971050?report=genbank&log$=nuclalign&blast_rank=1&RID=36M1KNZR015)) | **99** |
| 16 | 316 | *Shewanella litorisediminis* (γ-P, [NR_118420](https://www.ncbi.nlm.nih.gov/nucleotide/645321459?report=genbank&log$=nuclalign&blast_rank=3&RID=36M1KNZR015)) | **91** |
| 17 | 434 | *Novosphingobium malaysiense* (α-P, [NR_126280](https://www.ncbi.nlm.nih.gov/nucleotide/699005411?report=genbank&log$=nuclalign&blast_rank=1&RID=36M1KNZR015)) | **97** |
| 18 | 488 | *Salipiger nanhaiensis* (α-P, [NR_134804](https://www.ncbi.nlm.nih.gov/nucleotide/974142284?report=genbank&log$=nuclalign&blast_rank=1&RID=36M1KNZR015)) | **97** |
| 19 | 475 | *Salipiger nanhaiensis* (α-P, [NR_134804](https://www.ncbi.nlm.nih.gov/nucleotide/974142284?report=genbank&log$=nuclalign&blast_rank=1&RID=36M1KNZR015)) | **99** |
| 20 | 491 | *Mesoflavibacter sabulilitoris* (Fla, [NR_134082](https://www.ncbi.nlm.nih.gov/nucleotide/961555114?report=genbank&log$=nuclalign&blast_rank=1&RID=36M1KNZR015)) | **99** |
| 21 | 465 | *Parvibaculum indicum* (α-P, [NR_116565](https://www.ncbi.nlm.nih.gov/nucleotide/636560505?report=genbank&log$=nuclalign&blast_rank=1&RID=36E3JWYT015)) | **99** |
| 22 | 433 | *Thioprofundum hispidum* (γ-P, [NR_112620](https://www.ncbi.nlm.nih.gov/nucleotide/631251423?report=genbank&log$=nuclalign&blast_rank=1&RID=36M1KNZR015)) | **93** |
| 23 | 538 | *Pantoea agglomerans* (γ-P, [NR_041978](https://www.ncbi.nlm.nih.gov/nucleotide/343201252?report=genbank&log$=nuclalign&blast_rank=2&RID=36K2R44E015)) | **99** |
| 24 | 461 | *Amnibacterium soli* (Act, [NR_135734](https://www.ncbi.nlm.nih.gov/nucleotide/1011034848?report=genbank&log$=nuclalign&blast_rank=1&RID=36E3JWYT015)) | **95** |
| 25 | 479 | *Salinimicrobium xinjiangense* (Fla, [NR_044260](https://www.ncbi.nlm.nih.gov/nucleotide/343198991?report=genbank&log$=nuclalign&blast_rank=1&RID=36E3JWYT015)) | **98** |
| 26 | 409 | *Thioprofundum hispidum* (γ-P, [NR_112620](https://www.ncbi.nlm.nih.gov/nucleotide/631251423?report=genbank&log$=nuclalign&blast_rank=1&RID=36M1KNZR015)) | **92** |
| 27 | 480 | *Amnibacterium soli* (Act, [NR_135734](https://www.ncbi.nlm.nih.gov/nucleotide/1011034848?report=genbank&log$=nuclalign&blast_rank=1&RID=36E3JWYT015)) | **99** |
| 28 | 354 | *Alcanivorax gelatiniphagus* (γ-P, [NR_136483](https://www.ncbi.nlm.nih.gov/nucleotide/1018196604?report=genbank&log$=nuclalign&blast_rank=1&RID=36M1KNZR015)) | **90** |
| 29 | 491 | *Alcanivorax dieselolei* (γ-P, [NR_043106](https://www.ncbi.nlm.nih.gov/nucleotide/343202692?report=genbank&log$=nuclalign&blast_rank=4&RID=36E3JWYT015)) | **99** |
| Seawater sample from Kuwait Towers | | | |
| **1** | 344 | *Owenweeksia hongkongensis* (Fla, [NR_040990](https://www.ncbi.nlm.nih.gov/nucleotide/343200303?report=genbank&log$=nuclalign&blast_rank=3&RID=36E3JWYT015)) | 96 |
| **2** | 499 | *Brevibacterium frigoritolerans* (Bac, [NR_117474](https://www.ncbi.nlm.nih.gov/nucleotide/645320215?report=genbank&log$=nuclalign&blast_rank=1&RID=36K2R44E015)) | 100 |
| **3** | 439 | *Bacillus circulans* (Bac, [NR_118445](https://www.ncbi.nlm.nih.gov/nucleotide/645321491?report=genbank&log$=nuclalign&blast_rank=2&RID=36K2R44E015)) | 96 |
| **4** | 425 | *Pantoea agglomerans* (γ-P, [NR_041978](https://www.ncbi.nlm.nih.gov/nucleotide/343201252?report=genbank&log$=nuclalign&blast_rank=2&RID=36K2R44E015)) | 99 |
| **5** | 479 | *Salinimicrobium xinjiangense* (Fla, [NR_044260](https://www.ncbi.nlm.nih.gov/nucleotide/343198991?report=genbank&log$=nuclalign&blast_rank=1&RID=36E3JWYT015)) | 98 |
| **6** | 470 | *Altererythrobacter oceanensis* (α-P, [NR_134693](https://www.ncbi.nlm.nih.gov/nucleotide/974142173?report=genbank&log$=nuclalign&blast_rank=1&RID=36K2R44E015)) | 99 |
| **7** | 412 | *Marinobacter lipolyticus* (γ-P, [NR_025671](https://www.ncbi.nlm.nih.gov/nucleotide/219846081?report=genbank&log$=nuclalign&blast_rank=2&RID=36E3JWYT015)) | 93 |
| **8** | 437 | *Gramella oceani* (Fla, [NR_134045](https://www.ncbi.nlm.nih.gov/nucleotide/961555077?report=genbank&log$=nuclalign&blast_rank=8&RID=36E3JWYT015)) | 96 |
| **9** | 439 | *Erythrobacter vulgaris* (α-P, [NR_043136](https://www.ncbi.nlm.nih.gov/nucleotide/343202716?report=genbank&log$=nuclalign&blast_rank=1&RID=36E3JWYT015)) | 97 |
| **10** | 315 | *Thalassospira australica* (α-P, [KU553304](https://www.ncbi.nlm.nih.gov/nucleotide/983964128?report=genbank&log$=nuclalign&blast_rank=1&RID=36E3JWYT015)) | 89 |
| **11** | 430 | *Parvibaculum lavamentivorans* (α-P, NR_029105) | 96 |
| **12** | 409 | *Polaribacter dokdonensis* (Fla, [NR_043456](https://www.ncbi.nlm.nih.gov/nucleotide/343202961?report=genbank&log$=nuclalign&blast_rank=1&RID=36E3JWYT015)) | 100 |
| **13** | 502 | *Pantoea brenneri* (γ-P, [NR_116748](https://www.ncbi.nlm.nih.gov/nucleotide/636560688?report=genbank&log$=nuclalign&blast_rank=2&RID=36K2R44E015)) | 99 |
| **14** | 508 | *Alteromonas australica* (γ-P, [NR_116737](https://www.ncbi.nlm.nih.gov/nucleotide/636560677?report=genbank&log$=nuclalign&blast_rank=2&RID=36E3JWYT015)) | 99 |
| **15** | 503 | *Alteromonas australica* (γ-P, [NR_116737](https://www.ncbi.nlm.nih.gov/nucleotide/636560677?report=genbank&log$=nuclalign&blast_rank=2&RID=36E3JWYT015)) | 99 |
| **16** | 474 | *Thalassospira australica* (α-P, [KU553304](https://www.ncbi.nlm.nih.gov/nucleotide/983964128?report=genbank&log$=nuclalign&blast_rank=1&RID=36E3JWYT015)) | 100 |
| **17** | 387 | *Thiohalophilus thiocyanatoxydans* (γ-P, [NR_043875](https://www.ncbi.nlm.nih.gov/nucleotide/343198845?report=genbank&log$=nuclalign&blast_rank=1&RID=36E3JWYT015)) | 92 |
| **18** | 479 | *Novosphingobium malaysiense* (α-P, [NR_126280](https://www.ncbi.nlm.nih.gov/nucleotide/699005411?report=genbank&log$=nuclalign&blast_rank=1&RID=36K2R44E015)) | 99 |
| **19** | 451 | *Ponticaulis koreensis* (α-P, [NR_044608](https://www.ncbi.nlm.nih.gov/nucleotide/343206038?report=genbank&log$=nuclalign&blast_rank=1&RID=36K2R44E015)) | 98 |
| **20** | 396 | *Clostridium caminithermale* (Bac, [NR_041887](https://www.ncbi.nlm.nih.gov/nucleotide/343198454?report=genbank&log$=nuclalign&blast_rank=1&RID=36K2R44E015)) | 94 |
| **21** | 434 | *Ruegeria scottomollicae* (α-P, NR_042675) | 96 |
| 22 | 476 | *Parvibaculum indicum* (α-P, [NR_116565](https://www.ncbi.nlm.nih.gov/nucleotide/636560505?report=genbank&log$=nuclalign&blast_rank=1&RID=36E3JWYT015)) | 100 |
| 23 | 408 | *Neptuniibacter halophilus* (γ-P, [NR_108534](https://www.ncbi.nlm.nih.gov/nucleotide/566084996?report=genbank&log$=nuclalign&blast_rank=1&RID=36K2R44E015)) | 94 |
| 24 | 492 | *Alteromonas australica* (γ-P, [NR_116737](https://www.ncbi.nlm.nih.gov/nucleotide/636560677?report=genbank&log$=nuclalign&blast_rank=2&RID=36E3JWYT015)) | 99 |
| 25 | 471 | *Parvibaculum indicum* (α-P, [NR_116565](https://www.ncbi.nlm.nih.gov/nucleotide/636560505?report=genbank&log$=nuclalign&blast_rank=1&RID=36E3JWYT015)) | 99 |
| 26 | 478 | *Vibrio azureus* (γ-P, [NR_041683](https://www.ncbi.nlm.nih.gov/nucleotide/343200996?report=genbank&log$=nuclalign&blast_rank=4&RID=36E3JWYT015)) | 98 |
| 27 | 329 | *Ensifer adhaerens* (α-P, [NR_113893](https://www.ncbi.nlm.nih.gov/nucleotide/631252695?report=genbank&log$=nuclalign&blast_rank=5&RID=36K2R44E015)) | 89 |
| 28 | 494 | *Idiomarina zobellii* (γ-P, [KM407766](https://www.ncbi.nlm.nih.gov/nucleotide/831434884?report=genbank&log$=nuclalign&blast_rank=5&RID=36K2R44E015)) | 99 |
| 29 | 490 | *Thalassospira australica* (α-P, [KU553304](https://www.ncbi.nlm.nih.gov/nucleotide/983964128?report=genbank&log$=nuclalign&blast_rank=1&RID=36E3JWYT015)) | 98 |
| 30 | 358 | *Thiohalophilus thiocyanatoxydans* (γ-P, [NR_043875](https://www.ncbi.nlm.nih.gov/nucleotide/343198845?report=genbank&log$=nuclalign&blast_rank=1&RID=36E3JWYT015)) | 91 |
| 31 | 485 | *Parvibaculum indicum* (α-P, [NR_116565](https://www.ncbi.nlm.nih.gov/nucleotide/636560505?report=genbank&log$=nuclalign&blast_rank=1&RID=36E3JWYT015)) | 100 |
| 32 | 475 | *Maricaulis virginensis* (α-P, [NR_025444](https://www.ncbi.nlm.nih.gov/nucleotide/219878305?report=genbank&log$=nuclalign&blast_rank=1&RID=36K2R44E015)) | 99 |
| Seawater sample from Khiran | | | |
| **1** | 499 | *Gramella echinicola* (Fla, [NR_113919](https://www.ncbi.nlm.nih.gov/nucleotide/631252721?report=genbank&log$=nuclalign&blast_rank=1&RID=36E3JWYT015)) | 99 |
| **2** | 492 | *Limnobacter thiooxidans* (β-P, [NR_025421](https://www.ncbi.nlm.nih.gov/nucleotide/219878282?report=genbank&log$=nuclalign&blast_rank=1&RID=36E3JWYT015)) | 99 |
| **3** | 471 | *Erythrobacter vulgaris* (α-P, [NR_043136](https://www.ncbi.nlm.nih.gov/nucleotide/343202716?report=genbank&log$=nuclalign&blast_rank=1&RID=36E3JWYT015)) | 99 |
| **4** | 332 | *Thalassospira australica* (α-P, [KU553304](https://www.ncbi.nlm.nih.gov/nucleotide/983964128?report=genbank&log$=nuclalign&blast_rank=1&RID=36E3JWYT015)) | **89** |
| **5** | 411 | *Alcanivorax dieselolei* (γ-P, [NR_043106](https://www.ncbi.nlm.nih.gov/nucleotide/343202692?report=genbank&log$=nuclalign&blast_rank=4&RID=36E3JWYT015)) | **99** |
| **6** | 431 | *Gramella echinicola* (Fla, [NR_113919](https://www.ncbi.nlm.nih.gov/nucleotide/631252721?report=genbank&log$=nuclalign&blast_rank=1&RID=36E3JWYT015)) | **96** |
| **7** | 385 | *Marinobacter lipolyticus* (γ-P, [NR_025671](https://www.ncbi.nlm.nih.gov/nucleotide/219846081?report=genbank&log$=nuclalign&blast_rank=2&RID=36E3JWYT015)) | 97 |
| **8** | 400 | *Aliifodinibius roseus* (Sph, [NR_118428](https://www.ncbi.nlm.nih.gov/nucleotide/645321470?report=genbank&log$=nuclalign&blast_rank=1&RID=36E3JWYT015)) | **93** |
| **9** | 359 | *Aliifodinibius roseus* (Sph, [NR_118428](https://www.ncbi.nlm.nih.gov/nucleotide/645321470?report=genbank&log$=nuclalign&blast_rank=1&RID=36E3JWYT015)) | **91** |
| **10** | 466 | *Polaribacter dokdonensis* (Fla, [NR_043456](https://www.ncbi.nlm.nih.gov/nucleotide/343202961?report=genbank&log$=nuclalign&blast_rank=1&RID=36E3JWYT015)) | 98 |
| **11** | 510 | *Vibrio neptunius* (γ-P, [NR_025476](https://www.ncbi.nlm.nih.gov/nucleotide/219878337?report=genbank&log$=nuclalign&blast_rank=2&RID=36E3JWYT015)) | 100 |
| **12** | 310 | *Sphingomonas daechungensis* (α-P, [NR_133862](https://www.ncbi.nlm.nih.gov/nucleotide/959495011?report=genbank&log$=nuclalign&blast_rank=1&RID=36E3JWYT015)) | **89** |
| **13** | 486 | *Alteromonas australica* (γ-P, [NR_116737](https://www.ncbi.nlm.nih.gov/nucleotide/636560677?report=genbank&log$=nuclalign&blast_rank=2&RID=36E3JWYT015)) | 99 |
| **14** | 486 | *Alteromonas australica* (γ-P, [NR_116737](https://www.ncbi.nlm.nih.gov/nucleotide/636560677?report=genbank&log$=nuclalign&blast_rank=2&RID=36E3JWYT015)) | 99 |
| **15** | 518 | *Alteromonas australica* (γ-P, [NR_116737](https://www.ncbi.nlm.nih.gov/nucleotide/636560677?report=genbank&log$=nuclalign&blast_rank=2&RID=36E3JWYT015)) | 99 |
| **16** | 469 | *Oceanicola marinus* (α-P, [NR_043969](https://www.ncbi.nlm.nih.gov/nucleotide/343198900?report=genbank&log$=nuclalign&blast_rank=1&RID=36E3JWYT015)) | 99 |
| **17** | 454 | *Thalassospira australica* (α-P, [KU553304](https://www.ncbi.nlm.nih.gov/nucleotide/983964128?report=genbank&log$=nuclalign&blast_rank=1&RID=36E3JWYT015)) | 100 |
| 18 | 417 | *Lutibacter aestuarii* (Fla, [NR_108995](https://www.ncbi.nlm.nih.gov/nucleotide/566085191?report=genbank&log$=nuclalign&blast_rank=1&RID=36E3JWYT015)) | 96 |
| 19 | 511 | *Parvibaculum indicum* (α-P, [NR_116565](https://www.ncbi.nlm.nih.gov/nucleotide/636560505?report=genbank&log$=nuclalign&blast_rank=1&RID=36E3JWYT015)) | 99 |
| 20 | 395 | *Oceanibacterium hippocampi* (α-P, [NR_117037](https://www.ncbi.nlm.nih.gov/nucleotide/645319662?report=genbank&log$=nuclalign&blast_rank=1&RID=36E3JWYT015)) | 92 |
| 21 | 475 | *Pseudoalteromonas undina* (γ-P, [NR_114191](https://www.ncbi.nlm.nih.gov/nucleotide/631252993?report=genbank&log$=nuclalign&blast_rank=2&RID=36E3JWYT015)) | 98 |
| 22 | 353 | *Owenweeksia hongkongensis* (Fla, [NR_040990](https://www.ncbi.nlm.nih.gov/nucleotide/343200303?report=genbank&log$=nuclalign&blast_rank=3&RID=36E3JWYT015)) | 90 |
| 23 | 499 | *Idiomarina piscisalsi* (γ-P, [NR_132671](https://www.ncbi.nlm.nih.gov/nucleotide/926663077?report=genbank&log$=nuclalign&blast_rank=5&RID=36E3JWYT015)) | 99 |
| 24 | 441 | *Vibrio azureus* (γ-P, [NR_041683](https://www.ncbi.nlm.nih.gov/nucleotide/343200996?report=genbank&log$=nuclalign&blast_rank=4&RID=36E3JWYT015)) | 95 |
| 25 | 402 | *Desulforhabdus amnigena* (δ-P, [NR_029289](https://www.ncbi.nlm.nih.gov/nucleotide/265678981?report=genbank&log$=nuclalign&blast_rank=1&RID=36E3JWYT015)) | 93 |
| 26 | 415 | *Litorivivens lipolytica* (γ-P, [KM017973](https://www.ncbi.nlm.nih.gov/nucleotide/667481450?report=genbank&log$=nuclalign&blast_rank=1&RID=36E3JWYT015)) | 93 |
| 27 | 460 | *Amnibacterium soli* (Act, [NR_135734](https://www.ncbi.nlm.nih.gov/nucleotide/1011034848?report=genbank&log$=nuclalign&blast_rank=1&RID=36E3JWYT015)) | 96 |
| 28 | 479 | *Salinimicrobium xinjiangense* (Fla, [NR_044260](https://www.ncbi.nlm.nih.gov/nucleotide/343198991?report=genbank&log$=nuclalign&blast_rank=1&RID=36E3JWYT015)) | 98 |
| 29 | 349 | *Cytophaga fermentans* (Bact, [NR_112979](https://www.ncbi.nlm.nih.gov/nucleotide/631251781?report=genbank&log$=nuclalign&blast_rank=1&RID=36E3JWYT015)) | 91 |
| 30 | 456 | *Marinobacterium litorale* (γ-P, [NR_044016](https://www.ncbi.nlm.nih.gov/nucleotide/343205652?report=genbank&log$=nuclalign&blast_rank=1&RID=36E3JWYT015)) | 95 |
| 31 | 398 | *Hyphomonas atlantica* (α-P, [KF863140](https://www.ncbi.nlm.nih.gov/nucleotide/633896647?report=genbank&log$=nuclalign&blast_rank=1&RID=36E3JWYT015)) | 93 |
| 32 | 398 | *Thiohalophilus thiocyanatoxydans* (γ-P, [NR_043875](https://www.ncbi.nlm.nih.gov/nucleotide/343198845?report=genbank&log$=nuclalign&blast_rank=1&RID=36E3JWYT015)) | 93 |
| 33 | 303 | *Halochromatium salexigens* (γ-P, [NR_036810](https://www.ncbi.nlm.nih.gov/nucleotide/310974946?report=genbank&log$=nuclalign&blast_rank=1&RID=36E3JWYT015)) | 91 |
| 34 | 461 | *Parvularcula lutaonensis* (α-P, [NR_044474](https://www.ncbi.nlm.nih.gov/nucleotide/343205959?report=genbank&log$=nuclalign&blast_rank=1&RID=36E3JWYT015)) | 97 |
| 35 | 428 | *Parvibaculum indicum* (α-P, [NR_116565](https://www.ncbi.nlm.nih.gov/nucleotide/636560505?report=genbank&log$=nuclalign&blast_rank=1&RID=36E3JWYT015)) | 100 |

α-P, α-Proteobacteria; β-P, β-Proteobacteria; γ-P, γ-Proteobacteria; δ-P, δ-Proteobacteria; Act, Actinobacteria; Fla, Flavobacteriia; Bac, Bacilli; Bact, Bacteroidia; Sph, Sphingobacteriia
